# 

**Published:** 1894-06

**Authors:** 


### Communicated.

### NORTH-WESTERN UNIVERSITY DENTAL SCHOOL.

The commencement exercises of the North-western Dental School were held April 24th. There were twenty-four graduates: Lucian Hauch Arnold, William Spencer Bagley, Samuel Augustus Bell, Edwin Kenton Remmington, Leander Read Brown. Louis Arthur Edwards, Charles Herbert Eldred, Benjamin Franklin Ellis, Frederick Wilhelm Fritsche, Robert Leslie Hopkins, Philip Freeman Kittor, Louis Ladewick, William Lansing Lewis, Fred. William Marriett, Paul George Maxon, Abraham Louis Oberndorf, Caspar Paul Plaff, Hosea Edwin Reid, Charles Mackay Smith, Edward Henry Smith, Claude Bryant Warner, Awrin Ralph Shaw, Herbert Devere Wilson, Charles Casper Lipe.

AMERICAN DENTAL ASSOCIATION.—The thirty-fourth Annual Session of the American Dental Association will be held at Old Point Comfort, commencing at 10 a. m. Tuesday August 7, 1894.

GEO. H. CUSHING,
Recording Secty.

SOUTHERN DENTAL ASSOCIATION.—The Southern Dental Association will hold its regular meeting this year at Old Point Comfort, Va., beginning Thursday, August 2nd, and continuing until Monday, August 6th inclusive. Liberal reductions in hotel and railroad fair have been secured, and the Virginia Association have promised some pleasant social surprises.

### To Readers and Correspondents.

Communications intended for publication, and exchange journals and papers, should be addressed to the Editor of The American Journal of Dental Science, No. 845 N. Eutaw St. Baltimore, Md. All letters relating to the business of the Journal, or containing cash remittances, to be directed to Messrs. Snowden & Cowman, No. 9 W. Fayette Street, Baltimore, Md. Articles for publication should be received by the Editor at least two weeks previous to the first of the month on which the number in which it is designed for them to appear, is issued.

☞ The American Journal of Dental Science will contain fifty pages of reading matter in each number, making a volume of about Six Hundred pages,

EXCLUSIVE OF ADVERTISEMENTS

